# *Leuconostoc mesenteroides* LVBH107 Antibacterial Activity against *Porphyromonas gingivalis* and Anti-Inflammatory Activity against *P. gingivalis* Lipopolysaccharide-Stimulated RAW 264.7 Cells

**DOI:** 10.3390/nu14132584

**Published:** 2022-06-22

**Authors:** Chang Luan, Jiaqing Yan, Ning Jiang, Chuang Zhang, Xu Geng, Zhengqiang Li, Chen Li

**Affiliations:** 1 Key Laboratory for Molecular Enzymology and Engineering of the Ministry of Education, College of Life Sciences, Jilin University, Changchun 130012, China; luanchang6789@163.com (C.L.); yjq@mail.jlu.edu.cn (J.Y.); zhangchuang19@mails.jlu.edu.cn (C.Z.); gengxu21@mails.jlu.edu.cn (X.G.); 2 State Key Laboratory for Zoonotic Diseases, Key Laboratory for Zoonosis Research of the Ministry of Education, Institute of Zoonosis, College of Veterinary Medicine, Jilin University, Changchun 130062, China; jiangning19@mails.jlu.edu.cn

**Keywords:** *Leuconostoc mesenteroides*, antibacterial, *Porphyromonas gingivalis*, anti-inflammatory, macrophage

## Abstract

Probiotics, active microorganisms benefiting human health, currently serve as nutritional supplements and clinical treatments. Periodontitis, a chronic infectious oral disease caused by *Porphyromonas gingivalis* (*P. gingivalis*), activates the host immune response to release numerous proinflammatory cytokines. Here, we aimed to clarify *Leuconostoc mesenterica* (*L. mesenteroides*) LVBH107 probiotic effects based on the inhibition of *P.* *gingivalis* activities while also evaluating the effectiveness of an in vitro *P.* *gingivalis* lipopolysaccharide-stimulated RAW 264.7 cell-based inflammation mode. *L. mesenteroides* LVBH107 survived at acid, bile salts, lysozyme, and hydrogen peroxide conditions, auto-aggregated and co-aggregated with *P. gingivalis*, exhibited strong hydrophobicity and electrostatic action, and strongly adhered to gingival epithelial and HT-29 cells (thus exhibiting oral tissue adherence and colonization abilities). Moreover, *L.* *mesenteroides* LVBH107 exhibited sensitivity to antibiotics erythromycin, doxycycline, minocycline, ampicillin, and others (thus indicating it lacked antibiotic resistance plasmids), effectively inhibited *P.* *gingivalis* biofilm formation and inflammation (in vitro inflammation model), reduced the secretion of pro-inflammatory cytokines (TNF-α, IL-6 and IL-1β) and inflammatory mediators (NO and PGE_2_), and decreased the expression levels of inflammation related genes. Thus, *L.* *mesenterica* LVBH107 holds promise as a probiotic that can inhibit *P.* *gingivalis* biofilm formation and exert anti-inflammatory activity to maintain oral health.

## 1. Introduction

Probiotics are defined by the FAO/WHO as “live microorganisms which when administered in adequate amounts confer a health benefit on the host” [1]. The effectiveness of probiotics is strain-specific, such that different strains appear to influence host health through different mechanisms. In recent years, probiotics that improve the host microecological balance have been used to promote health based on their abilities to adjust microbial community structure and regulate the immune-inflammatory response [2,3,4]. Several clinical studies have confirmed that probiotics can alleviate intestinal diseases (diarrhea, constipation, irritable bowel syndrome), metabolic diseases (diabetes, hyperlipidemia, cardiovascular and cerebrovascular diseases), immune-related diseases (chronic enteritis, asthma, allergy), and neurological diseases (autism, depression, Alzheimer’s disease) [5,6,7,8]. Although activities of probiotics have been frequently reported to improve host intestinal barrier integrity and modulate the host’s innate and adaptive immune responses [9], probiotic effects on oral health, including dental diseases, have been rarely reported [10].

Periodontitis is a common chronic inflammatory oral disease that is caused by oral pathogenic microbes [11], which is an immune response to pathogen invasion. In the absence of antimicrobial treatment, periodontic inflammation leads to periodontal tissue destruction that triggers an exaggerated immune response that can destroy the periodontal ligament and lead to alveolar bone loss [12]. Macrophages are precursors of osteoclasts, which appear in the early stage of periodontitis and mediate alveolar bone loss. M1 macrophages mainly secrete inflammatory cytokines; increasing the number of M1 macrophages or the proportion of M1/M2 macrophages can promote the progression of periodontitis [13]. Current treatments for periodontitis involve intensive and expensive therapies, such as scaling and root planing, as well as antibiotic treatments that do not prevent disease and can promote antibiotic resistance. Due to drawbacks associated with current treatments, a more appealing natural and non-invasive method to treat oral disease is sought that would replace antibiotics. Toward this end, researchers have begun to explore certain *Lactobacillus* and *Lactococcus* species as prospective probiotic treatments for oral diseases [14]. In an early study investigating treatment of gum tissues with *Lactobacillus reuteri* (*L. reuteri*), Tweetman et al. [15] found markedly lower levels of TNF-α and interleukin-8 (IL-8) in the gingival crevicular fluid of patients with periodontal disease versus corresponding levels in healthy controls. Moreover, clinical trials have shown that the administration of probiotics containing *Lactobacillus brevis* to patients with periodontal disease led to decreased gingivitis severity and dental calculus deposition [16], while results of another study demonstrated that gingivitis was significantly alleviated in patients who took probiotic preparations containing *L. reuteri* [17]. Furthermore, Lauritano et al. [18] demonstrated the improved clinical efficacy of probiotics used as adjuvant treatments for alleviating peri-implant inflammation, as reflected by the post-treatment improvement of patient gingival index values and decreased numbers of periodontal pathogens. Taken together, the results of these studies suggest that probiotics can be used to treat diseases affecting oral health.

*Porphyromonas gingivalis* (*P. gingivalis*) is currently recognized as an important pathogen involved in mature dental plaque formation from biofilm generated through activities of a variety of associated pathogenic or nonpathogenic bacteria that adhere to and copolymerize with *P. gingivalis* cells. In addition, a variety of virulence factors (including fimbriae, capsules, lipopolysaccharides, proteases, and toxic metabolites) that participate in biofilm formation are expressed during interactions of these bacteria with host cells [19], whereby bacteria can bind to and trigger host Toll-like receptor signaling. After these receptors are triggered, they stimulate the host cell production of cytokines with different biological functions that mediate the anti-microbial immune response, including an inflammatory cascade response that is thought to be associated with periodontitis [20,21]. Therefore, the inhibition of *P. gingivalis* by the use of probiotic organisms holds promise as an important approach for preventing and slowing the progression of destructive periodontal disease.

*Leuconostoc mesenteroides* (*L. mesenteroides*), a Gram-positive, spherical or short-chain facultative anaerobic lactobacillus, is widely used for the production of fermented foods and bacteriocins. Several studies have shown that *L. mesenteroides* treatments can exert antioxidant activities, improve immunity, reduce cholesterol levels, and alleviate hyperlipidemia [22,23]. For example, John et al. reported that *L. mesenteroides* administration could effectively reduce levels of the inflammatory factor IL-6, prompting researchers to speculate that *L. mesenteroides* may serve as a safe immunomodulatory treatment [24]. In our previous study, bacteria of the potential probiotic strain *L. mesenteroides* LVBH107 were isolated from a traditional fermented food (spicy cabbage) and shown to exert an obvious inhibitory effect on *P. gingivalis* activities as based on Oxford cup assay results (Table S1). Those results led us to hypothesize that *L. mesenteroides* LVBH107 could be used to prevent or alleviate *P. gingivalis* lipopolysaccharide (LPS)-stimulated inflammation, prompting the current study. Here, potential probiotic properties of *L. mesenteroides* LVBH107 were investigated in vitro, including resistance, surface characteristics, antibiotic resistance, and anti-*P. gingivalis* activity, assessed inflammatory responses of *L. mesenteroides* LVBH107 using a model of inflammation based on *P. gingivalis* LPS-stimulated RAW 264.7 macrophage. Thereby, our results provide experimental and theoretical basis for further using LVBH107 as an auxiliary drug to inhibit periodontal pathogens and treat periodontitis.

## 2. Materials and Methods

### 2.1. Bacterial Strains, Culture Conditions, and Sample Preparation

*Leuconostoc mesenteroides* (subsp. *mesenteroides*) LVBH107 (CGMCC No. 21362) that obtained from a fermented food product (spicy cabbage) were deposited in the China General Microbiological Culture Collection Center (Beijing, China). *L. mesenteroides* LVBH107 that was inoculated (3%, *v*/*v*) into de Man, Rogosa, and Sharpe (MRS) broth (Hopebio Company, Qingdao, China) was cultured in an anaerobic incubator (85% N_2_, 10% H_2_, and 5% CO_2_) at 37 °C for 16 h.

After culture, *L. mesenteroides* LVBH107 was collected by centrifugation (1500× *g*, 10 min at 4 °C); then, the pellet was washed three times with phosphate-buffered saline (PBS and adjusted to 9 Log CFU/mL before the organisms were used in experiments. Meanwhile, *L. mesenteroides* LVBH107 culture medium was centrifuged (1500× *g*, 10 min at 4 °C) to obtain the bacteria-free supernatant, which was filtered through a 0.22 μM filter membrane to remove bacterial cells. In addition, a heat-killed *L. mesenteroides* LVBH107 preparation was prepared by heating the bacteria at 90 °C for 30 min.

*P. gingivalis* (ATCC 3327) was obtained from the China General Microbiological Culture Collection Center and was propagated on Columbia blood agar (Hopebio Company, Qingdao, China) at 37 °C in an anaerobic incubator (85% N_2_, 10% H_2_, and 5% CO_2_) for 48 h. Next, brain heart infusion (BHI) broth (Hopebio Company) was inoculated with a *P. gingivalis* culture (3%, *v*/*v*) followed by incubation at 37 °C for 48 h.

### 2.2. Cell Culture Conditions

Human colorectal adenocarcinoma HT-29, human gingival epithelial (HGE), and RAW 264.7 (mouse mononuclear macrophage-derived) cell lines were obtained from Otwo Biotech (Shenzhen, China) and cultured in Dulbecco’s Modified Eagle Medium (DMEM; HyClone, Logan, UT, USA) containing 10% (*v*/*v*) fetal bovine serum (FBS; HyClone) and 100 U/mL Penicillin-Streptomycin (Sigma-Aldrich, St. Louis, MO, USA) at 37 °C (5% CO_2_, 90% relative humidity). Cells were subcultured once every 2 days by splitting cultures 1:3 in fresh medium after harvesting adherent cells by detaching them from flask surfaces using 0.25% (*w*/*v*) trypsin-EDTA solution (Sigma-Aldrich).

### 2.3. Probiotic Properties of L. mesenteroides LVBH107

#### 2.3.1. Pepsin, Bile, Lysozyme, and Hydrogen Peroxide Tolerance

An overnight culture of *L. mesenteroides* LVBH107 was inoculated at 3% (*v*/*v*) into MRS broth containing 0.3% (*w*/*v*) pepsin (pH 2.0, pH 3.0, or pH 5.0), bovine bile (0.3% or 0.5%, *w*/*v*), lysozyme (100 μg/mL or 200 μg/mL, *w*/*v*), and hydrogen peroxide (H_2_O_2_, 0.08 mM or 0.8 mM, *w*/*v*); then, cultures were incubated at 37 °C for 20 h under aerobic conditions. During the culture period, optical density (OD) values at 600 nm were measured using a UV-VIS spectrophotometer (UV-2700, Shimadzu, Kyoto, Japan) every 2 h.

#### 2.3.2. Auto-Aggregation Ability

After overnight culture, *L. mesenteroides* LVBH107 cells were collected by centrifugation (1500× *g*, 10 min at 4 °C) and resuspended in PBS. The OD_600_ of the suspension was adjusted to 1.0 using a UV-VIS spectrophotometer; then, the suspension was incubated at 37 °C for 8 h, during which OD_600_ values were measured at 2 h, 4 h, 6 h, and 8 h [25]. Auto-aggregation ability was calculated as follows:

Auto-aggregation ability (%) = (1 − A_T_/A_0_) × 100%

where A_T_ is the OD_600_ value at 2 h, 4 h, 6 h, and 8 h, and A_0_ is the OD_600_ value at 0 h. 

#### 2.3.3. Co-Aggregation Ability

After *L. mesenteroides* LVBH107 and *P. gingivalis* strains were harvested at 16 h or 48 h by centrifugation (1500× *g*, 10 min at 4 °C), cells of each strain were resuspended in sterile PBS; then, the OD_600_ values of the two strains were adjusted to 0.50 and 0.60, respectively. Next, equal volumes (2 mL) of suspensions of both strains were shaken (200 r/min, 5 min), and each suspension was separately incubated without shaking for 8 h at 37 °C. During the 8 h incubation, OD_600_ readings of each suspension were taken at 2 h, 4 h, 6 h, and 8 h [26]. Co-aggregation ability was calculated as follows:

Co-aggregation ability (%) = [((A_X_ + A_Y_)/2 − A_mix_)/(A_X_ + A_Y_)/2] × 100%

where A_X_ and A_Y_ are absorbance values of *L. mesenteroides* LVBH107 and *P. gingivalis* at 0 h and A_mix_ is the absorbance value of the mixture containing both organisms after incubation for 2 h, 4 h, 6 h, and 8 h.

#### 2.3.4. Surface Hydrophobicity

The surface hydrophobicity of *L. mesenteroides* LVBH107 was determined using the microbial adhesion to hydrocarbons (MATH) method with toluene and xylene serving as hydrophobic organic solvents. Chloroform was selected as Lewis acid and ethyl acetate as Lewis base in order to determine surface charge characteristics of *L. mesenteroides* LVBH107 [27]. The method used for *L. mesenteroides* LVBH107 preparation was the same method as described above except that the bacterial pellet was resuspended in 0.1 M KNO_3_ buffer (pH 6.2) and adjusted to an OD_600_ value of 0.60. Next, 1 mL of organic solvent (toluene, xylene, ethyl acetate, and chloroform) to 3 mL of the *L. mesenteroides* LVBH107 suspension were combined to form a two-phase solution that was preincubated at room temperature for 10 min then vortexed for 2 min followed by incubation at room temperature for 30 min. Thereafter, the OD_600_ value of the aqueous phase was determined; then, the adherence of *L. mesenteroides* LVBH107 to hydrocarbons was calculated as follows:

Cell surface hydrophobicity (%) = [1 − A_1_/A_0_] × 100%

where A_0_ is the OD_600_ value before treatment with the organic solvent and A_1_ is the OD_600_ value of the aqueous phase after treatment with the organic solvent. 

#### 2.3.5. Adhesion Ability

HT-29 and HGE cells were transferred to 12-well tissue culture plate wells (1 × 10^5^ cells/well); then, the plates were incubated until cells adhered completely to well surfaces and reached 70–80% confluence. Next, medium in the wells was replaced with high glucose DMEM medium (without penicillin-streptomycin); then, the plates were incubated at 37 °C for 24 h. Thereafter, monolayers of cells were washed three times with PBS; then, 500 μL of *L. mesenteroides* LVBH107 (8 Log CFU/mL) was added per well, and then the plates were incubated for 2 h at 37 °C. Next, non-adherent bacteria were removed from each well then 2 mL of 1% (*v*/*v*) Triton X-100 (Sigma-Aldrich Co., St. Louis, MO, USA) was added to each well to detach adherent cells [28]; then, a flat colony counting method was used to detect the numbers of viable bacteria. The adhesion ability of the *L. mesenteroides* LVBH107 was calculated as follows:

Survival rate (%) = V_1_/V_0_ × 100%

where V_1_ is the total count of adhered *L. mesenteroides* LVBH107, and V_0_ is the number of total added *L. mesenteroides* LVBH107.

#### 2.3.6. Safety Assessment

The antibiotic susceptibility of *L. mesenteroides* LVBH107 was determined using the disk diffusion method. First, 1 mL of *L. mesenteroides* LVBH107 (8 Log CFU/mL) culture was evenly spread onto the surface of each newly prepared MRS agar plate after the agar solidified at room temperature. Next, commercial antibiotic disks were placed onto plate surfaces, and then the plates were incubated at 37 °C for 48 h. After incubation, the diameter of each inhibition zone around each disk was measured and expressed as susceptible (S), intermediate (I), or resistant (R), as specified within the instructions provided by the manufacturer (BKMAM, Changde, China). All analyses were performed in triplicate.

### 2.4. Antibacterial Activity of L. mesenteroides LVBH107 against P. gingivalis

#### 2.4.1. Biofilm Formation Assay

*P. gingivalis* cultures that had been incubated for 48 h at 37 °C were adjusted to 6 Log CFU/mL by dilution in BHI broth. Next, *L. mesenteroides* LVBH107 culture medium or supernatant and *P. gingivalis* were added to upper and lower chambers of 24-well transwell plates (0.4 μm, Corning, NY, USA), respectively, at a ratio of 1:1 (*v*/*v*). After the incubation of transwell plates for 48 h at 37 °C, the fluid culture medium within the lower chamber was gently removed and discarded; then, the *P. gingivalis* biofilm at the bottom of each well was gently washed three times with PBS to remove planktonic *P. gingivalis*. Thereafter, *P. gingivalis* biofilm in each lower chamber was fixed in 2.5% (*v*/*v*) glutaraldehyde solution at 4 °C overnight and dried at room temperature. Next, 100 μL of 0.1% (*w*/*v*) crystal violet was added to each lower transwell chamber; then, staining was allowed to proceed at room temperature for 15 min, after which wells that had received stain were rinsed gently with 75% ethanol to remove unbound crystal violet and dried at room temperature [29]. Thereafter, *P. gingivalis* biofilm structure was observed under a microscope (IX73, Olympus, Kyoto, Japan) and photographed then biofilm absorbance values were measured at 540 nm using a microplate reader. The rate of *L. mesenteroides* LVBH107 inhibition of *P. gingivalis* biofilm formation was calculated as follows:

Biofilm inhibition rate (%) = [1 − A_S_/A_C_] × 100%

where AS is the OD_540_ value of *P. gingivalis* biofilm in the absence of *L. mesenteroides* LVBH107 culture medium or supernatant, and AC is the OD_540_ value of the *P. gingivalis* biofilm treated with *L. mesenteroides* LVBH107 culture medium or supernatant.

#### 2.4.2. Biofilm Activity Assay

After sterilized coverslips (10 mm × 10 mm) were placed into lower chambers of transwell plates, co-cultures containing *L. mesenteroides* LVBH107 culture medium or supernatant and *P. gingivalis* were prepared in transwell plates as described above. After incubation of transwell plates for 72 h at 37 °C, coverslips coated with *P. gingivalis* biofilm were stained for 15-20 min in the dark using a LIVE/DEAD^®^ BacLight™ Bacterial Viability Kit (L7012, Thermo Fisher Scientific, Waltham, MA, USA) [30]. Next, *P. gingivalis* biofilm structure on each coverslip was assessed and recorded using a confocal laser scanning microscope (CLSM, LSM710, Carl Zeiss, Oberkochen, Germany).

### 2.5. Assessment of Functional Properties of L. mesenteroides LVBH107 Using the RAW 264.7 Cell Model

#### 2.5.1. Cytotoxicity Assay

The cytotoxicity of *L. mesenteroides* LVBH107 for RAW 264.7 cells was assessed using the Cell Counting Kit-8 (CCK-8) assay (APExBIO, Houston, TX, USA). After RAW 264.7 cells were inoculated into wells of 96-well plates and allowed to adherently grow on well surfaces to 70–80% confluence (1 × 10^6^ cells/well), different concentrations of live or heat-killed *L. mesenteroides* LVBH107 were added to the wells (0, 7, 8, and 9 Log CFU/mL) followed by co-incubation of plates for 6 h at 37 °C. Thereafter, the culture medium was removed from the wells; then, 300 μL of CCK-8 working reagent was added to each well, followed by the incubation of plates at 37 °C for 4 h. Next, the optical density of each well at 450 nm was measured using a multi-function microplate reader (F200 Pro, Tecan Infinite, Männedorf, Switzerland). Cell viability was calculated as follows:

Cell viability (%) = As/Ac × 100%

where As is the OD_450_ value of RAW 264.7 cells co-incubated with *L. mesenteroides* LVBH107, and Ac is the OD_450_ value of RAW 264.7 cells in the absence of *L. mesenteroides* LVBH107.

#### 2.5.2. Cytokine and Inflammation Assays

To assess *P. gingivalis* anti-inflammatory activity, RAW 264.7 cells were treated with live or heat-killed *L. mesenteroides* LVBH107 suspensions for 3 h or 6 h and then 1 μg/mL of *P. gingivalis* LPS (InvivoGen, San Diego, CA, USA) was added to cells followed by incubation of stimulated cells for 24 h (37 °C, 5% CO_2_, 90% relative humidity). Next, the production of nitric oxide (NO) was determined in *P. gingivalis* LPS-stimulated RAW 264.7 cells using assay kits to measure levels of each compound (Nanjing Jiancheng Bioengineering Institute, Nanjing, China). In addition, the effects of *L. mesenteroides* LVBH107 on the production of TNF-α, interleukin-6 (IL-6), interleukin-1β (IL-1β), and prostaglandin E_2_ (PGE_2_) were assessed in *P. gingivalis* LPS-induced RAW 264.7 cells using enzyme linked immunosorbent assay (ELISA) kits (Jianglai Industrial Company, Shanghai, China) [31].

#### 2.5.3. RNA Extraction and Quantitative Real-Time Polymerase Chain Reaction (qRT-PCR) Assay

RAW 264.7 cells were transferred to centrifuge tubes; then, total RNA was extracted according to instructions provided with the TaKaRa MiniBEST Universal RNA Extraction Kit (TaKaRa, Kyoto, Japan). Next, the Primescript™ RT reagent Kit (TaKaRa) was used to reverse transcribe approximately 1.0 μg of total RNA into cDNA [32]. The cDNA served as template for semi-quantitative real-time PCR to determine relative transcriptional expression levels of genes encoding *TNF-α*, *IL-6*, *IL-1β*, cyclooxygenase-2 (*COX-2*), and inducible nitric oxide synthase (*iNOS*). The 2^−ΔΔCt^ method was used to calculate qRT-PCR results. Melting curve analysis was used to evaluate the specificity of the reaction. *β-actin* served as the internal control. Primer sequences are listed in Table 1.

### 2.6. Statistical Analysis

Data are provided as mean ± standard deviation. All experiments were repeated three times. All statistical analyses were performed using SPSS 22.0 (Chicago, IL, USA) software. Significant differences between groups were assessed using one-way ANOVA and LSD post hoc analysis [33]. Values of *p* < 0.05 were considered statistically significant.

## 3. Results

### 3.1. Probiotic Characterization of L. mesenteroides LVBH107

**Figure 1:**
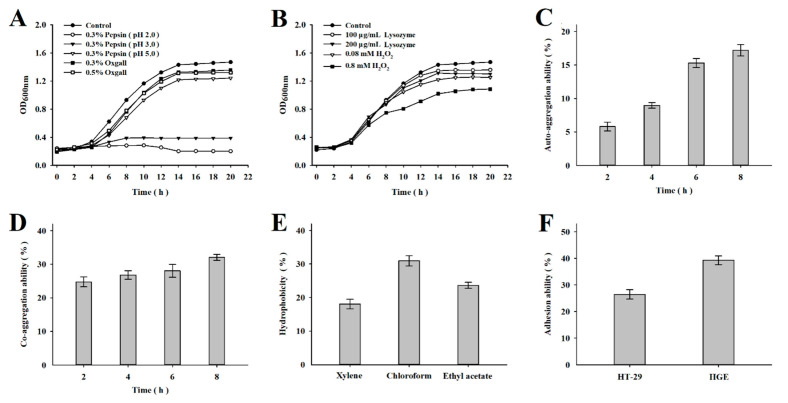
Probiotic characteristics of *L. mesenteroides* LVBH107. (**A**) Growth curves of *L. mesenteroides* LVBH107 exposed to MRS broth contained 0.3% pepsin (pH 2.0, pH 3.0, pH 5.0) and bovine bile salt (0.3%, 0.5%) for 20 h. (**B**) Growth curves of *L. mesenteroides* LVBH107 exposed to MRS broth contained lysozyme (100 μg/mL, 200 μg/mL) and H_2_O_2_ (0.08 mM, 0.8 mM) for 20 h. (**C**) Auto-aggregation ability of *L. mesenteroides* LVBH107. (**D**) Co-aggregation ability of *L. mesenteroides* LVBH107. (**E**) Surface hydrophobicity of *L. mesenteroides* LVBH107. (**F**) Adhesion ability of *L. mesenteroides* LVBH107 to HT-29 and HGE.

In order to function within the oral environment of the human host, probiotic organisms must possess strong tolerance to harsh conditions found there in order to remain viable and carry out activities that benefit human health. Figure 1A presents results demonstrating that *L. mesenteroides* LVBH107 survived and exhibited high growth activity after culture in 0.3% pepsin (pH 5.0) and bovine bile salts (0.3% and 0.5%) for 20 h, while in 0.3% pepsin at pH 2.0 and 3.0, the organisms grew slowly and survived only for as long as 10 h. In addition, *L. mesenteroides* LVBH107 (Figure 1B) exhibited excellent tolerance and survival when exposed to lysozyme (100 μg/mL, 200 μg/mL) and H_2_O_2_ (0.08 mM, 0.8 mM).

During the initial 2 h, a low rate of *L. mesenteroides* LVBH107 auto-aggregation (5.80%) was observed (Figure 1C), which increased significantly from 2 to 8 h and reached a rate of 17.19% at 8 h. As shown in Figure 1D, the co-aggregation rate of *L. mesenteroides* LVBH107 gradually increased from 2 to 8 h as *L. mesenteroides* LVBH107 organisms agglutinated with *P. gingivalis* organisms then reached a rate at 8 h of 32.05%.

Hydrophobicity results for *L. mesenteroides* LVBH107 are shown in Figure 1E, with hydrophobicity rates of *L. mesenteroides* LVBH107 to xylene, chloroform, and ethyl acetate found to be 18.06%, 30.95%, and 23.64%, respectively. These results indicate that cell surfaces of this strain carry large numbers of charges that can generate electrostatic forces. In order to understand adhesion ability of *L. mesenteroides* LVBH107, the adhesion rate of *L. mesenteroides* LVBH107 to HT-29 cells was determined experimentally and was found to be 26.45% (Figure 1F). In order to further evaluate the adhesion ability of *L. mesenteroides* LVBH107 to cells within the oral cavity, the adhesion of bacterial cells of this strain to HGE cells was also investigated, with the results showing an adhesion rate of 39.27%.

Next, the sensitivity of *L. mesenteroides* LVBH107 to 18 antibiotics was investigated using the agar diffusion method according to the Clinical and Laboratory Standards Institute 2017 guidelines, with test results shown in Table 2. *L. mesenteroides* LVBH107 was found to be sensitive to erythromycin, doxycycline, minocycline, ampicillin, chloramphenicol, and cefuroxime, resistant to penicillin, gentamicin, streptomycin, polymyxin B, vancomycin, rifampicin, cefazolin, and ceftazidime, and moderately sensitive to tetracycline, piperacillin, cefoperazone, and ceftriaxone. These results indicated that *L. mesenteroides* LVBH107 was susceptible to numerous antibiotics.

### 3.2. Inhibitory Effect of L. mesenteroides LVBH107 on P. gingivalis Biofilm Formation

#### 3.2.1. Inhibitory Effect of *L. mesenteroides* LVBH107 on Biofilm Formation by *P. gingivalis*

**Figure 2:**
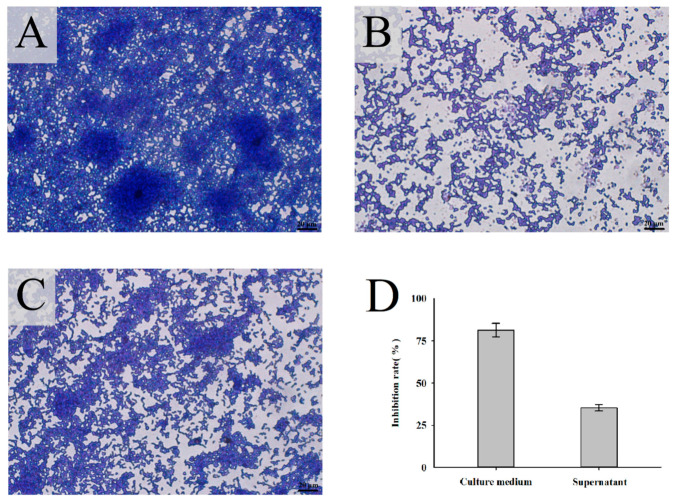
Microscopy images of *P. gingivalis* biofilm treated with *L. mesenteroides* LVBH107. *P. gingivalis* biofilm stained with crystal violet after cultured for 48 h. (**A**) Untreated control. (**B**) Treatment with *L. mesenteroides* LVBH107 culture medium. (**C**) Treatment with the supernatant of *L. mesenteroides* LVBH107. (**D**) Inhibition rate of *L. mesenteroides* LVBH107 on biofilm formation of *P. gingivalis*. Scale bar = 20 μm.

The growth characteristics of *P. gingivalis* biofilm stained with crystal violet were observed under a microscope. The results obtained for the control group (after *P. gingivalis* culture for 48 h) revealed a highly organized and uneven biofilm structure containing a large number of *P. gingivalis* organisms (Figure 2A). In addition, several mushroom-like microcolonies were observed within the control group biofilm. By contrast, the addition of *L. mesenteroides* LVBH107 culture medium to the *P. gingivalis* culture led to the significant inhibition of *P. gingivalis* biofilm formation and looser biofilm structure that lacked a membrane as compared to control biofilm (Figure 2B). Meanwhile, the supernatant of *L. mesenteroides* LVBH107 inhibited the aggregation of *P. gingivalis* biofilm (Figure 2C), resulting in *P. gingivalis* bacteria assuming a more loose arrangement as small microcolonies within an unevenly distributed biofilm containing large gaps. Furthermore, culture medium and bacteria-free culture supernatant of *L. mesenteroides* LVBH107 inhibited formation of biofilm at rates of 81.31% and 35.28% (Figure 2D), respectively, thus demonstrating that culture medium inhibition of biofilm formation was significantly greater than inhibition by culture supernatant.

#### 3.2.2. Inhibitory Effect of *L. mesenteroides* LVBH107 on Biofilm Structure

**Figure 3:**
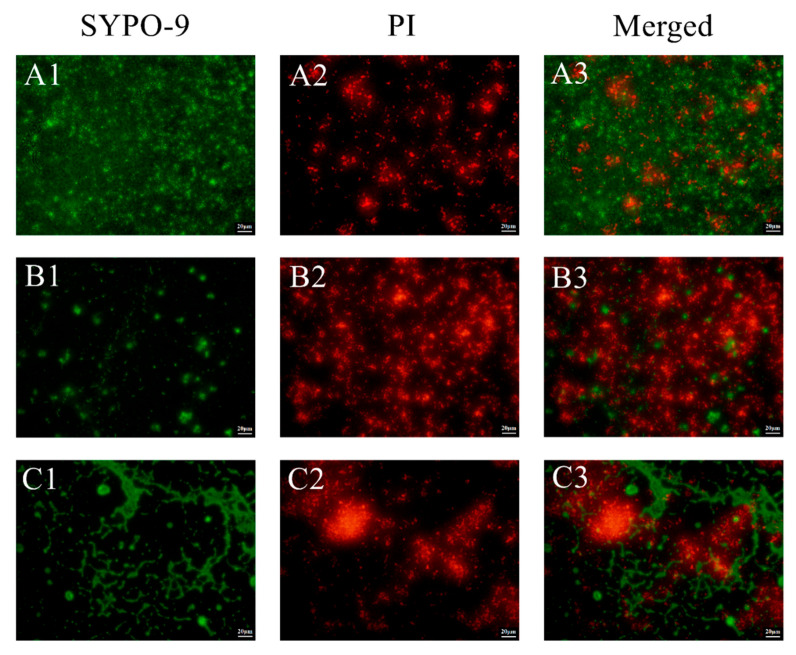
CLSM images of *P. gingivalis* biofilm treated with *L. mesenteroides* LVBH107. Live strains were stained green, whereas dead strains were stained red using the LIVE/DEAD^®^ BacLight™ Bacterial Viability kit (L7012, Thermo Fisher Scientific, Waltham, MA, USA) (**A**) Untreated control. (**B**) Treatment with *L. mesenteroides* LVBH107 culture medium. (**C**) treatment with the supernatant of *L. mesenteroides* LVBH107. (1) Stained with green fluorescence (SYTO9). (2) Stained with red fluorescence (PI). (3) Indicated merge of red and green fluorescence. Scale bar = 20 μm.

Adhesion and aggregation of *P. gingivalis* within biofilm that occurred during culture of the bacteria for 72 h were observed by CLSM. After fluorescent staining, live and dead bacteria were detected based on green and red staining of cells, respectively. As shown in Figure 3, the *P. gingivalis* biofilm of the control group mainly exhibited green fluorescence (Figure 3(A3)), indicating that lots of live *P. gingivalis* were present within the biofilm structure (Figure 3(A1)). After the addition of *L. mesenteroides* LVBH107 culture medium to *P. gingivalis* cultures, the *P. gingivalis* biofilm was observed to be sparse and loosely formed and unable to form a complete membrane structure (Figure 3(B3)). In addition, *P. gingivalis* cells within the biofilm were present in a scattered distribution, with increased numbers of cells exhibiting red fluorescence (dead bacteria) and decreased numbers of cells exhibiting green fluorescence (live bacteria), as shown in Figure 3(B1,B2). In fact, the *P. gingivalis* biofilm generally stained red, indicating that most cells had died (Figure 3(B2,B3)). The addition of *L. mesenteroides* LVBH107 supernatant to *P. gingivalis* cultures also led to the disruption and destruction of the biofilm structure, with a portion of the biofilm exhibiting red fluorescence (dead *P. gingivalis* cells) and a portion exhibiting green fluorescence (live *P. gingivalis* cells), as shown in Figure 3(C1–C3). Therefore, our results demonstrated that *L. mesenteroides* LVBH107 could effectively inhibit *P. gingivalis* biofilm formation, with the *L. mesenteroides* LVBH107 culture medium inhibitory effect found to be greater than of the bacteria-free culture supernatant.

### 3.3. In Vitro Anti-Inflammatory Properties of L. mesenteroides LVBH107

#### 3.3.1. Effect of *L. mesenteroides* LVBH107 on RAW 264.7 Cells

**Figure 4:**
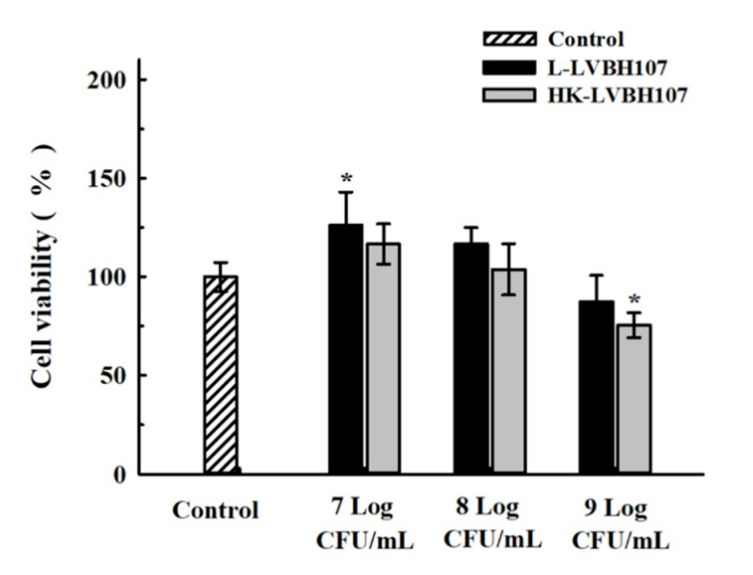
Effect of *L. mesenteroides* LVBH107 on the cell viability of RAW264.7 cells. * *p* < 0.05, compared to control group. Actual *p*-values are shown in Table S2.

In order to assess potential cytotoxic effects of *L. mesenteroides* LVBH107 when the strain was added to RAW 264.7 macrophages in vitro, we evaluated the viability of RAW 264.7 macrophage-like cells when exposed to different *L. mesenteroides* LVBH107 concentrations. As shown in Figure 4, additions of 7 Log CFU/mL and 8 Log CFU/mL *L. mesenteroides* LVBH107 to RAW 264.7 cells were associated with obvious greater RAW 264.7 cell viability (to varying degrees) as compared to the control group without observed cytotoxic effects, with cell viability reaching 103.76–126.45% after 6 h co-culture with *L. mesenteroides* LVBH107. Importantly, the addition of live *L. mesenteroides* LVBH107 to RAW 264.7 cells achieved a significantly greater effect than was achieved by the addition of heat-killed bacteria. By contrast, under the same conditions as mentioned above, the addition of 9 Log CFU/mL bacteria to RAW 264.7 cells led to decreases in RAW 264.7 cell viability and proliferation. These results suggest that the cytotoxic effect induced by the addition of 9 Log CFU/mL *L. mesenteroides* LVBH107 resulted in a partial cell death that ultimately led to reduced viable cell numbers. Therefore, 7 Log CFU/mL and 8 Log CFU/mL concentrations of both live and heat-killed *L. mesenteroides* LVBH107 were used in subsequent experiments.

#### 3.3.2. Anti-Inflammatory of *L. mesenteroides* LVBH107 on Proinflammatory Cytokines Expression in *P. gingivalis* LPS-Induced RAW 264.7 Cells

**Figure 5:**
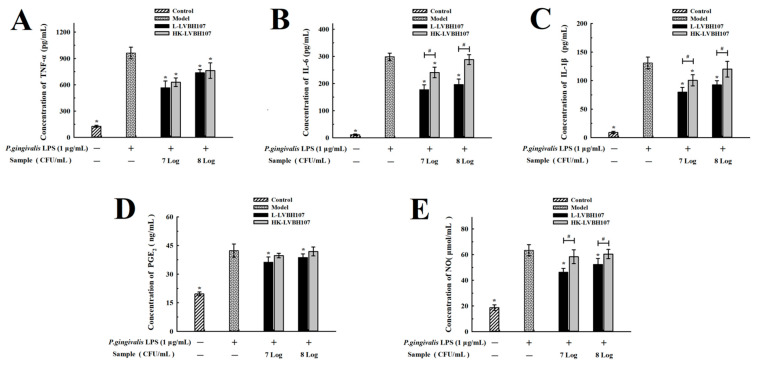
Effect of *L. mesenteroides* LVBH107 on proinflammatory cytokines in RAW264.7 cells. RAW264.7 cells were treated with live or heat-killed *L. mesenteroides* LVBH107 and stimulated with *P. gingivalis* LPS for 24 h. The levels of TNF-α (**A**), IL-6 (**B**), and IL-1β (**C**) were assessed with ELISA kits. The levels of PGE_2_ (**D**) and NO (**E**) were assessed with assay kits. * *p* < 0.05, compared to *P. gingivalis* LPS group; ^#^ *p* < 0.05, comparison between live and heat-killed *L. mesenteroides* LVBH107. Actual *p*-values are shown in Tables S2–S7.

As expected, the exposure of RAW 264.7 cells to *P. gingivalis* LPS for 24 h clearly stimulated the cells and led to increased levels of proinflammatory cytokines (TNF-α, IL-6, and IL-1β). However, the treatment of RAW 264.7 cells with *L. mesenteroides* LVBH107 suppressed levels of secreted TNF-α, IL-6, and IL-1β to varying degrees after *P. gingivalis* LPS stimulated (Figure 5A–C), thus alleviating the RAW 264.7 cell inflammatory state. As compared with the *P. gingivalis* LPS model group, secreted proinflammatory cytokine levels of RAW 264.7 cells exposed to a low dose (7 Log CFU/mL) of *L. mesenteroides* LVBH107 were significantly reduced after *P. gingivalis* LPS stimulation, while the exposure of these RAW 264.7 cells to heat-killed *L. mesenteroides* LVBH107 led to a reduced inhibition of proinflammatory cytokine secretion than was observed after addition of live *L. mesenteroides* LVBH107 to RAW 264.7 cells.

*P. gingivalis* LPS stimulation also significantly increased the secretion levels of inflammatory mediators PGE_2_ and NO in macrophage RAW264.7 (Figure 5D,E). Compared with the *P. gingivalis* LPS model group, secreted PGE_2_ levels of RAW 264.7 cells exposed to 7 Log CFU/mL and 8 Log CFU/mL of *L. mesenteroides* LVBH107 were reduced to varying degrees after *P. gingivalis* LPS stimulation (Figure 5D). Additionally, the inhibitory effect of live *L. mesenteroides* LVBH107 on the PGE_2_ secretion of RAW 264.7 cells was more obvious. Similarly, after the exposure of macrophages RAW264.7 to *L. mesenteroides* LVBH107, NO secretion was significant inhibited (Figure 5E). Moreover, live *L. mesenteroides* LVBH107 exhibited more obvious inhibitory effect on the secretion of inflammatory mediators compared to heat-killed *L. mesenteroides* LVBH107 at concentrations of 7 Log CFU/mL and 8 Log CFU/mL.

#### 3.3.3. Effect of *L. mesenteroides* LVBH107 on mRNA Expression Levels in *P. gingivalis* LPS-Induced RAW 264.7 Cells

**Figure 6:**
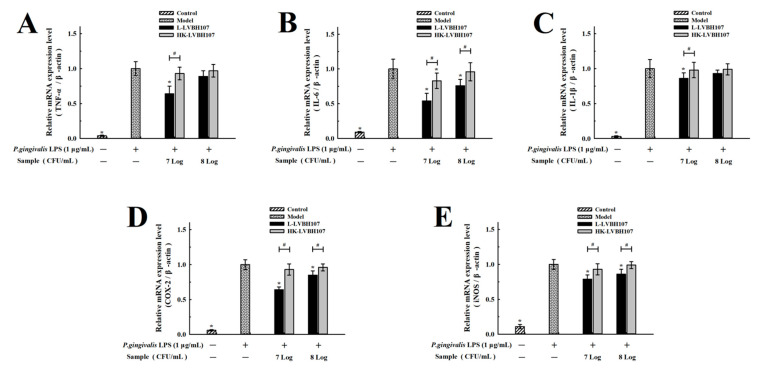
Effect of *L. mesenteroides* LVBH107 on mRNA expression in *P. gingivalis* LPS-stimulated RAW264.7 cells. RAW264.7 cells were treated with live or heat-killed L. mesenteroides LVBH107 and stimulated with *P. gingivalis* LPS for 24 h. Expressed mRNA levels of *TNF-α* (**A**), *IL-6* (**B**), *IL-1β* (**C**), *COX-2* (**D**), and *iNOS* (**E**) were determined with relative quantification by normalization with β-actin. * *p* < 0.05, compared to *P. gingivalis* LPS group; ^#^ *p* < 0.05, comparison between live and heat-killed *L. mesenteroides* LVBH107. Actual *p*-values are shown in Tables S8–S12.

After mRNA-level expression levels of pro-inflammatory cytokines (*TNF-α*, *IL-6*, and *IL-1β*) were up-regulated by LPS stimulation, the treatment of RAW 264.7 cells with *L. mesenteroides* LVBH107 led to relative down-regulated expression of these cytokines (Figure 6A–C). Similarly, mRNA levels corresponding to *COX-2* and *iNOS* expression were also significantly decreased (Figure 6D,E). Importantly, similar inhibitory effects were observed when heat-killed *L. mesenteroides* LVBH107 were added to RAW 264.7 cells in place of live *L. mesenteroides* LVBH107.

## 4. Discussion

As a probiotic that must exert its beneficial activity within the oral cavity, lactobacillus must be able to survive within the harsh oral environment and adhere to oral mucosal cells for a certain period of time. Indeed, our results demonstrated that *L. mesenteroides* LVBH107 exhibited acid and bile salts tolerance (Figure 1A) in addition to tolerance to lysozyme and H_2_O_2_ that were found within the oral microenvironment, whereby *L. mesenteroides* LVBH107 could tolerate 0.8 mM H_2_O_2_ and 200 μg/mL lysozyme (Figure 1B) while maintaining high viability and proliferative activity under these conditions. Furthermore, these results thus suggest that *L. mesenteroides* LVBH107 possesses characteristics that support its survival within the extreme environment, as consistent with results reported by Angmo et al. and Chul et al. [34,35], indicating that probiotics such as *Lactobacillus plantarum (L. plantarum)* and *Lactobacillus fermentans (L. fermentans)* could survive under conditions with low pH, bile salts, and lysozyme.

Importantly, a previous study demonstrated that lactobacillus strains with strong auto-aggregation ability could colonize mucosal surfaces and consequently carry out their probiotic functions better than strains without this ability [36]. Notably, *L. mesenteroides* LVBH107 was confirmed here to possess an auto-aggregation ability (Figure 1C) that gradually increased with prolongation of cell–cell contact time. Meanwhile, a strong co-aggregation ability has been shown to help probiotic organisms remove cells of other bacterial species from the oral cavity [37], thus reducing mouth colonization by pathogenic bacteria. Consequently, here, we investigated *L. mesenteroides* LVBH107 co-aggregation with *P. gingivalis* and observed good co-aggregation ability between cells of these strains. Here, we must point out that bacterial colonization is related to cell surface hydrophobicity, an important property that can influence the strength of an interaction between lactic acid bacteria and the environment or host. In fact, the results of a previously reported study demonstrated that higher bacterial surface hydrophobicity strengthened the bacteria–host interaction by enhancing bacterial adherence to host epithelial cells [36,37]. In this study, *L. mesenteroides* LVBH107 cells exhibited a high level of hydrophobicity, as evidenced by high bacterial cell adhesion to chloroform and ethyl acetate, in alignment with results obtained in other studies with regard to surface properties of *Lactobacillus* and *Streptococcus* [38] and results reported in another study demonstrating that adhesion ability was required for bacteria to exert a probiotic effect in the host [39]. In addition, the reported results obtained using in vitro HT-29 and HGE cell-based model systems revealed high adhesion rates of selected lactobacillus strains to both types of cells, with *L. mesenteroides* LVBH107 exhibiting a higher HT-29 cell adhesion rate than that of *Lactobacillus rhamnosus* LGG (6.1%) [40]. Meanwhile, the adhesion and invasion of host tissues have been found to be key steps in the pathogenesis of a variety of pathogenic bacteria and viruses [41], with studies confirming that adherent lactobacillus strains competitively blocked adhesion between pathogens and host cells to effectively inhibit pathogen invasion and host cell binding [42]. Therefore, we speculated that *L. mesenteroides* LVBH107, through auto-aggregation and co-aggregation activities, may form a barrier that protects the host epithelium by blocking binding of potential pathogens to host cell receptors.

Due to the fact that antibiotic genes encoded by plasmids can be transferred among bacterial strains [43], this activity may be a risk factor associated with probiotics use. For that reason, antibiotic sensitivity is an important factor that may be used to assess the safety of potential probiotics. Notably, De Almeida et al. and Essid et al. [44,45] reported that most lactobacillus strains were sensitive to tetracycline and chloramphenicol, as consistent with results obtained in the current study showing that *L. mesenteroides* LVBH107 possessed good biosafety characteristics (lacked antibiotic resistance plasmids), as reflected by its sensitivity to erythromycin, doxycycline, minocycline, ampicillin, chloramphenicol, and cefuroxime. However, here, we must note that tolerance to antibiotics can differ depending on species of lactobacillus under study and even among isolates of a given strain obtained from different sources. For example, Tulumoglu et al. [46] reported that 90% of lactobacillus strains were resistant to gentamicin, while a separate investigation of 17 strains of *L. plantarum* isolated from traditional cured meat in Tunisia revealed that most of them were resistant to rifampicin [45]. Moreover, *L. plantarum* isolates obtained from traditional Marseille dairy products were sensitive to penicillin [47], a result that was inconsistent with results of the current study.

*P. gingivalis*, the main pathogenic bacterial species that has been reported to be associated with chronic periodontitis, has been detected at a high rate (60.9%) within subgingival tissues of patients with periodontitis [48]. In fact, the colonization of periodontal tissues with *P. gingivalis* and subsequent dental plaque formation are main pathogenic factors associated with periodontitis. Dental plaque, a type of bacterial biofilm, is a soft, nonmineralized bacterially derived material that sticks to teeth and covers tooth surfaces. Importantly, dental plaque provides ideal attachment sites that support the colonization and growth of oral pathogens that play key roles in the pathogenesis of periodontitis. Therefore, inhibiting dental plaque biofilm formation is an important way to prevent periodontitis. In the present study, *L. mesenterica* LVBH107 significantly inhibited formation of *P. gingivalis* biofilm (Figure 2), whereby the addition of *L. mesenterica* LVBH107 culture medium to *P. gingivalis* cultures significantly inhibited *P. gingivalis* aggregation and led to the formation of an incomplete biofilm structure (Figure 2B). Meanwhile, cell-free *L. mesenterica* LVBH107 supernatant inhibited *P. gingivalis* biofilm formation and resulted in the formation of a biofilm that was unevenly populated with microcolonies separated by large gaps (Figure 2C). It has been reported that probiotics such as *L. fermentans* and *L. reuteri* could effectively inhibit activities of oral pathogens and reduce biofilm production [49,50,51], prompting this study to assess the unknown inhibitory effects of *L. mesenterica* on *P. gingivalis* biofilm.

Biofilm activities can be observed quickly and intuitively using confocal laser scanning microscopy (CLSM) and fluorescent staining. Two commonly used fluorescent stains include SYTO9, which can enter all bacterial cells to produce green fluorescence, and propidium iodide (PI), which can only enter bacterial cells with damaged cell membranes to produce red fluorescence. Here, CLSM results revealed that *L. mesenterica* LVBH107 could not only reduce the number of bacteria within a biofilm but could also affect bacterial survival status. After the treatment of *P. gingivalis* cultures with *L. mesenterica* LVBH107 culture medium, *P. gingivalis* biofilm structural formation was inhibited, the number of *P. gingivalis* within the biofilm was decreased, higher biofilm levels of red fluorescence were detected, and the proportion of living bacteria within the biofilm was decreased (which corresponded to an increase in the proportion of dead bacteria). Notably, biofilm damage increased after the addition of cell-free *L. mesenterica* LVBH107 culture supernatant to *P. gingivalis* cultures, with some *P. gingivalis* organisms found to be dead (as detected based on red fluorescence) and some found to be alive (as detected based on green fluorescence). Previously reported observations indicate the protection of microorganisms within biofilm due to the encapsulation of organisms by LPS, peptidoglycan, gingival protease, and flagellin that were produced via plaque biofilm metabolic activities [52]. Taken together, the abovementioned results suggest that *L. mesenterica* LVBH107 may inhibit growth of *P. gingivalis* and biofilm formation by inhibiting *P. gingivalis* activities within the biofilm, which ultimately reduces the production of extracellular products that support *P. gingivalis* survival within the oral microenvironment.

Macrophages are important immune system cells that play key roles in various inflammatory processes [53]. During the early stage of a classical inflammatory response, the pathogen triggers the innate immune response, resulting in macrophage activation followed by polarization that leads to the development of M1 macrophages. Once activated, M1 macrophages participate in pathogen elimination by releasing enzymes (iNOS, and COX-2), inflammatory mediators (NO, PGE_2_), reactive oxygen species, proinflammatory cytokines (TNF-α, IL-6, IL-1β, etc.), and cell chemokines [54] that support pathogen clearance via inflammatory processes. After pathogen clearance is complete, macrophages polarize and develop into M2 macrophages that down-regulate the expression and secretion of inflammatory mediators while also releasing anti-inflammatory cytokines such as TGF-β and IL-10 [55]. Importantly, the production of main inflammatory mediators NO, PGE_2_, iNOS, and COX-2 is regulated by upstream enzymes, with nitric oxide synthase (iNOS) acting as the rate-limiting enzyme that controls the NO synthesis pathway. In fact, the triggering of iNOS synthesis by inflammatory processes induces high-level expression and the release of large amounts of NO that, in turn, can combine with superoxide anion to form nitroso anions that play crucial roles in killing pathogenic microorganisms and tumors [56]. Meanwhile, COX-2, a rate-limiting enzyme of prostaglandin synthesis that is expressed rapidly after macrophages are stimulated by inflammation, generates PGs (especially PGE_2_) that participate in the inflammatory response [57]. PGE_2_ dilates blood vessels and lowers blood pressure, leading to up-regulated expression of other pro-inflammatory mediators (including cytokines) that may cause severe cell damage resulting from inflammatory disease processes [58]. Here, as expected, we observed increased NO and PGE_2_ levels in *P. gingivalis* LPS-stimulated RAW 264.7 cells that were both decreased after treated with *L. mesenterica* LVBH107 due to *L. mesenterica* LVBH107 inhibition of mRNA-level expression of iNOS and COX-2. This result aligned with results of similar studies showing that heat-inactivated *Weissella cibaria* JW15 and *L. brevis* K65 bacteria could inhibit production of NO and PGE_2_ by down-regulating *iNOS* and *COX-2* mRNA-level expression [28,59]. However, those results differed from results obtained here, whereby results here revealed that live bacteria inhibited production of the mediators better than heat-killed bacteria.

Pro-inflammatory cytokines mainly include TNF-α, IL-6, and IL-1β, which are produced by macrophages and endothelial cells. IL-1β and TNF-α promote inflammatory cell aggregation and activation, stimulate the release of inflammatory mediators, stimulate fever production, and exacerbate inflammatory responses [60,61]. IL-6 can stimulate macrophages to secrete monocyte chemotaxis protein-1 (MCP-1), which promotes monocyte exudation from blood vessels into inflammatory sites within tissues [62]. These pro-inflammatory cytokines can also increase endothelial cell secretion of leukocyte adhesion factors that promote tight cell–cell adhesion that can aggravate endothelial injury. Therefore, reducing pro-inflammatory cytokines is very important for alleviation of inflammatory diseases. In this work, unstimulated RAW 264.7 cells secreted proinflammatory cytokines at very low levels, while *P. gingivalis* LPS activated the macrophage inflammatory response and significantly increased mRNA-level expression of pro-inflammatory cytokines *TNF-α*, *IL-6*, and *IL-1β*. By contrast, treated with *L. mesenterica* LVBH107 bacteria attenuated the RAW 264.7 cell inflammatory state by inhibiting mRNA-level expression of *TNF-α*, *IL-6*, and *IL-1β* to reduce the production of these pro-inflammatory factors. Therefore, these findings collectively suggest that *L. mesenterica* LVBH107 may function as an anti-inflammatory probiotic, as consistent with results of numerous other studies demonstrating anti-inflammatory effects of other probiotics, such as *Bifidobacterium*, *Lactobacillus paracei*, and *Lactobacillus swiss*, that inhibited the expression and/or secretion of pro-inflammatory cytokines TNF-α, IL-6, and IL-1β [63,64,65]. We speculate that *L. mesenterica* LVBH107 produced an extracellular metabolite that exerted an anti-inflammatory effect that inhibited TNF-α, IL-1β, and IL-8 production by specifically targeting an anti-inflammatory molecular pathway. Notably, a similar mechanism was reported previously for *L. mesenteroides* exopolysaccharides S81 and BioE-LMD18 [66,67], which exerted immunomodulatory effects by regulating production of pro- and anti-inflammatory cytokines.

## 5. Conclusions

**Figure 7:**
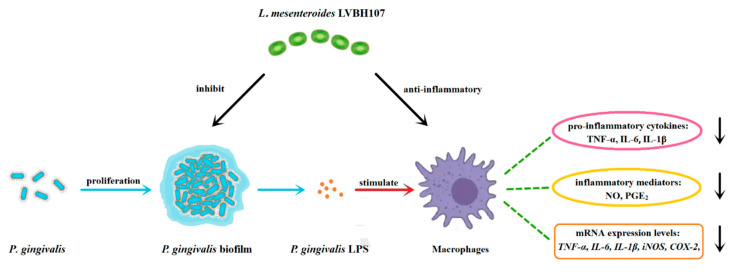
Schematic representation of the role for *L. mesenterica* LVBH107 in the inhibition of *P. gingivalis* and anti-inflammatory effectiveness of an in vitro *P. gingivalis* LPS-stimulated RAW 264.7 cell-based inflammation mode. LPS, lipopolysaccharide; TNF-α, tumor necrosis factor-α; IL-6, interleukin 6; IL-1β, interleukin 1β; NO, nitric oxide; PGE_2_, prostaglandin E_2_; COX-2, cyclooxygenase-2; iNOS, inducible nitric oxide synthase.

In summary, results of this work demonstrated antibacterial and anti-inflammatory effects of *L. mesenterica* LVBH107, a probiotic isolated from a traditional fermented food (spicy cabbage). This biosafe strain can survive in extreme environments, such as low-pH, bile salts, hydrogen peroxide, and lysozyme; at the same time, it has auto-aggregation, co-aggregation, and adhesion capacity abilities that encourage *L. mesenterica* LVBH107 adhesion and colonization of the oral environment. In vitro studies have shown that *L. mesenterica* LVBH107 effectively inhibited *P. gingivalis* biofilm formation, as well as bacterial activities within established biofilm. In addition, *L. mesenterica* LVBH107 attenuated the inflammatory response of *P. gingivalis* LPS-stimulated RAW 264.7 cells and reduced both expression and secretion of inflammatory mediators and pro-inflammatory factors, thus exhibiting anti-inflammatory potential (Figure 7). Taken together, our results suggest that *L. mesenterica* LVBH107 could serve as a highly promising probiotic with beneficial antibacterial and immunomodulatory activities, which could be one candidate for doctors in the treatment of periodontal inflammation and protecting teeth far from periodontal pathogen infection.

## Figures and Tables

**Table 1:** Nucleotide sequences of primers.

Gene	Primer Sequence (5′–3′)
*β-actin*	Forward: GTGGGCCGCCCTAGGCACCAG
	Reverse: GGAGGAAGAGGATGCGGCAGT
*TNF-α*	Forward: TTGACCTCAGCGCTGAGTTG
	Reverse: CCTGTAGCCCACGTCTAGC
*IL-6*	Forward: GTACTCCAGAAGACCAGAGG
	Reverse: TGCTGGTGACAACCACGGCC
*IL-1β*	Forward: CAGGATGAGGACATGAGCACC
	Reverse: CTCTGCAGACTCAAACTCCAC
*COX-2*	Forward: CACTACATCCTGACCCACTT
	Reverse: ATGCTCCTGCTTGAGTATGT
*iNOS*	Forward: CCCTTCCGAAGTTTCTGGCAGCAGC
	Reverse: GGCTGTCAGAGCCTCGTGGCTTTGG

**Table 2:** Antibiotic sensitivity of *L. mesenteroides* LVBH107.

Antimicrobial Agent		Disk Content (μg)	Criteria of Inhibition Zone Diameters (mm)			Detection Result	
Group	Drug		R	I	S	Inhibition Zone (mm)	Sensibility
macrolides	Erythromycin	15	≤15	16–20	≥21	28.2 ± 2.3	S
Tetracyclines	Doxycycline	30	≤12	13–15	≥16	19.9 ± 1.4	S
	Tetracycline	30	≤11	12–14	≥15	14.6 ± 0.6	I
	Minocyline	30	≤12	13–15	≥16	22.3 ± 2.4	S
β-Lactams Penicillins	Penicillin	10	≤18	-	≥29	24.5 ± 1.7	R
	Piperacillin	100	≤17	18–20	≥21	19.4 ± 0.9	I
	Ampicillin	10	≤11	12–14	≥15	15.4 ± 0.7	S
Aminoglycosides	Gentamicin	10	≤12	13–14	≥15	0	R
	Streptomycin	10	≤12	13–14	≥15	0	R
Lipopeptides	Polymyxin B	300	≤8	9–11	≥12	6.2 ± 1.1	R
Glycopeptides	Vancomycin	30	≤14	-	≥15	0	R
Chloramphenicol	Chloramphenicol	30	≤13	14–17	≥18	10.8 ± 1.0	R
ansamycins	Rifampicin	5	≤16	17–19	≥20	13.7 ± 0.8	R
Cephems	Cefazolin	30	≤19	20–22	≥23	10.1 ± 0.6	R
	Cefuroxime	30	≤14	15–22	≥23	21.6 ± 1.5	S
	Cefoperazone	75	≤15	16–20	≥21	19.5	I
	Ceftazidime	30	≤17	18–20	≥21	0	R
	Ceftriaxone	30	≤19	20–22	≥23	21.0	I

S—susceptible; I—intermediate; R—resistance.

## Data Availability

The data presented in this study are available in the article and its Supplementary Materials.

## Supplementary Materials

The following supporting information can be downloaded at: https://www.mdpi.com/article/10.3390/nu14132584/s1, Table S1: Antibacterial circle diameter of lactobacillus to Porphyromonas gingivalis; Table S2: Effect of *L. mesenteroides* LVBH107 on the cell viability of RAW264.7 cells.; Table S3: Effect of *L. mesenteroides* LVBH107 on TNF-α in RAW264.7 cells; Table S4: Effect of *L. mesenteroides* LVBH107 on IL-6 in RAW264.7 cells; Table S5: Effect of *L. mesenteroides* LVBH107 on IL-1β in RAW264.7 cells; Table S6: Effect of *L. mesenteroides* LVBH107 on IL-1β in RAW264.7 cells; Table S7: Effect of *L. mesenteroides* LVBH107 on PGE2 in RAW264.7 cells.; Table S8: Effect of *L. mesenteroides* LVBH107 on TNF-α mRNA expression in RAW264.7 cells; Table S9: Effect of *L. mesenteroides* LVBH107 on IL-6 mRNA expression in RAW264.7 cells; Table S10: Effect of *L. mesenteroides* LVBH107 on IL-1β mRNA expression in RAW264.7 cells; Table S11: Effect of *L. mesenteroides* LVBH107 on COX-2 mRNA expression in RAW264.7 cells; Table S12: Effect of *L. mesenteroides* LVBH107 on iNOS mRNA expression in RAW264.7 cells.
