# Supplementary material for: Leuconostoc mesenteroides LVBH107 Antibacterial Activity against Porphyromonas gingivalis and Anti-Inflammatory Activity against P. gingivalis Lipopolysaccharide-Stimulated RAW 264.7 Cells
Source: Nutrients. 2022 Jun 22;14(13):2584. doi: 10.3390/nu14132584 (PMC9268581; doi:10.3390/nu14132584)
Supplement: Supplementary file 1 [file nutrients-14-02584-s001.zip › nutrients-1780532-supplementary.pdf]

Supplementary Materials

1. Preliminary screening of lactobacillus strain inhibiting *Porphyromonas gingivalis*

Oxford cup drilling method was used to screen probiotics for inhibiting *Porphyromonas gingivalis*. 200 μL 1×10^7 CFU/mL *Porphyromonas gingivalis* was evenly spread on Columbia blood agar plate, and 4 holes were drilled on the agar with Oxford cup. 100 μL MRS liquid medium (as a control group) and lactobacillus with different concentrations (1×10^8 CFU/mL, 1×10^7 CFU/mL, 1×10^6 CFU/mL) were added to holes, respectively. After the liquid was absorbed by solid medium, the plate was inverted in a 37℃ incubator for 48 h. Use vernier calipers to measure the diameter of the bacteriostatic zone under the concentrations of 1×10^8 CFU/mL.

Table S1. Antibacterial circle diameter of lactobacillus to *Porphyromonas gingivalis.*

Strain	Inhibition zone (mm)	Strain	Inhibition zone (mm)
Leuconostoc mesenteroides LVBH101	14.7	*Lactobacillus sakei* MS101	-
Leuconostoc mesenteroides LVBH107	28.9	*Lactobacillus sakei* MS107	16.9
Leuconostoc mesenteroides LVBH108	16.9	*Lactobacillus sakei* MS108	14.5
Lactobacillus curvatus LUA101	24.1	*Lactobacillus sakei* MS109	-
Lactobacillus curvatus LUA102	14.3	*Lactobacillus sakei* MS201	16.8
Lactobacillus curvatus LUA103	-	*Weissella cibaria* BSH507	17.4
Lactobacillus curvatus LUA104	-	*Weissella cibaria* BSH509	-
Lactobacillus curvatus LUA105	13.6	*Weissella cibaria* BSH510	19.4
Lactobacillus curvatus LUA206	18.9	*Weissella cibaria* CBN101	22.1
Lactobacillus curvatus LUA207	15.8	*Weissella cibaria* CBN102	20.5
Pediococcus pentosaceus BSF203	-	*Weissella cibaria* CBN203	20.4
Pediococcus pentosaceus BSF204	-	*Weissella cibaria* CBK101	19.8
Pediococcus pentosaceus BSF206	21.6	*Weissella cibaria* CBK102	16.4
Pediococcus pentosaceus BSF207	-	*Weissella cibaria* CBK103	22.4
Pediococcus pentosaceus BSF208	16.5	*Weissella cibaria* CBK104	19.4
Pediococcus pentosaceus AC1-1	17.8	*Lactobacillus plantarum* K203	18.9
Pediococcus pentosaceus AC1-2	21.7	*Lactobacillus plantarum* K204	-
Pediococcus pentosaceus AC2-1	16.4	*Lactobacillus plantarum* K205	17.8
Pediococcus pentosaceus AC2-2	22.1	*Lactobacillus plantarum* K206	18.9
Pediococcus pentosaceus AC2-3	22.9	*Lactobacillus plantarum* K207	14.8
Lactobacillus sakei BSA103	14.1	*Lactobacillus plantarum* K208	19.0
Lactobacillus sakei BSA206	20.9	*Lactobacillus plantarum* H102	-
Lactobacillus sakei MS103	16.8	*Lactobacillus plantarum* H103	-
Lactobacillus sakei MS104	23.7	*Lactobacillus plantarum* H104	20.9
Lactobacillus sakei MS105	16.5	*Lactobacillus plantarum* H105	16.9

2. Relevant experimental data and *p* values

Table S2. Effect of *L. mesenteroides* LVBH107 on the cell viability of RAW264.7 cells.

Group	mean ± standard deviation	*p*1	*p*2	*p*3	*p*4
Control	100.00±7.32				
7 Log-L LVBH10	126.50±16.34	0.011	0.288		
7 Log-HK LVBH1	116.50±10.16	0.090	0.288		
8 Log-L LVBH10	116.74±10.07	0.086		0.173	
8 Log-HK LVBH1	103.71±13.14	0.689		0.173	
9 Log-L LVBH107	87.08±13.39	0.176			0.220
9 Log-HK LVBH10	75.44±6.26	0.017			0.220

*p*1 is the *p* value compared with the control group, *p*2 is the *p* value compared with 7-L LVBH107 or 7-HK LVBH107 group, *p*3 is the *p* value compared with 8-L LVBH107 or 8-HK LVBH107 group, *p*4 is the *p* value compared with 9-L LVBH107 or 9-HK LVBH107 group.

Table S3. Effect of *L. mesenteroides* LVBH107 on TNF-α in RAW264.7 cells.

Group	mean ± standard deviation	*p*1	*p*2	*p*3
Control	126.89±11.64	0.000		
Model	962.53±64.23			
7 Log-L LVBH107	565.88±76.67	0.000	0.220	
7 Log-HK LVBH107	629.24±49.35	0.000	0.220	
8 Log-L LVBH107	735.07±36.64	0.001		0.595
8 Log-HK LVBH107	761.81±87.46	0.001		0.595

*p*1 is the *p* value compared with the control group, *p*2 is the *p* value compared with 7-L LVBH107 or 7-HK LVBH107 group, *p*3 is the *p* value compared with 8-L LVBH107 or 8-HK LVBH107 group.

Table S4. Effect of *L. mesenteroides* LVBH107 on IL-6 in RAW264.7 cells.

Group	mean ± standard deviation	*p*1	*p*2	*p*3
Control	11.35±3.21	0.000		
Model	298.89±13.43			
7 Log-L LVBH107	176.38±18.18	0.000	0.000	
7 Log-HK LVBH107	241.48±19.13	0.010	0.000	
8 Log-L LVBH107	196.18±20.67	0.000		0.000
8 Log-HK LVBH107	287.9±18.40	0.433		0.000

*p*1 is the *p* value compared with the control group, *p*2 is the *p* value compared with 7-L LVBH107 or 7-HK LVBH107 group, *p*3 is the *p* value compared with 8-L LVBH107 or 8-HK LVBH107 group.

Table S5. Effect of *L. mesenteroides* LVBH107 on IL-1β in RAW264.7 cells.

Group	mean ± standard deviation	*p*1	*p*2	*p*3
Control	9.06±1.95	0.000		
Model	130.64±10.61			
7 Log-L LVBH107	79.4±8.67	0.000	0.019	
7 Log-HK LVBH107	100.22±9.58	0.002	0.019	
8 Log-L LVBH107	92.75±7.26	0.000		0.003
8 Log-HK LVBH107	120.88±14.01	0.228		0.003

*p*1 is the *p* value compared with the control group, *p*2 is the *p* value compared with 7-L LVBH107 or 7-HK LVBH107 group, *p*3 is the *p* value compared with 8-L LVBH107 or 8-HK LVBH107 group.

Table S6. Effect of *L. mesenteroides* LVBH107 on NO in RAW264.7 cells.

Group	mean ± standard deviation	*p*1	*p*2	*p*3
Control	18.77±2.25	0.000		
Model	63.87±4.26			
7 Log-L LVBH107	46.22±3.29	0.000	0.003	
7 Log-HK LVBH107	58.21±5.33	0.111	0.003	
8 Log-L LVBH107	52.28±4.67	0.040		0.030
8 Log-HK LVBH107	60.37±3.60	0.309		0.030

*p*1 is the *p* value compared with the control group, *p*2 is the *p* value compared with 7-L LVBH107 or 7-HK LVBH107 group, *p*3 is the *p* value compared with 8-L LVBH107 or 8-HK LVBH107 group.

Table S7. Effect of *L. mesenteroides* LVBH107 on PGE2 in RAW264.7 cells.

Group	mean ± standard deviation	*p*1	*p*2	*p*3
Control	19.63±1.00	0.000		
Model	42.39±3.43			
7 Log-L LVBH107	36.24±2.88	0.070	0.810	
7 Log-HK LVBH107	39.82±1.15	0.196	0.810	
8 Log-L LVBH107	38.6±1.96	0.067		0.111
8 Log-HK LVBH107	41.82±2.35	0.769		0.111

*p*1 is the *p* value compared with the control group, *p*2 is the *p* value compared with 7-L LVBH107 or 7-HK LVBH107 group, *p*3 is the *p* value compared with 8-L LVBH107 or 8-HK LVBH107 group.

Table S8. Effect of *L. mesenteroides* LVBH107 on *TNF-α* mRNA expression in RAW264.7 cells.

Group	mean ± standard deviation	*p*1	*p*2	*p*3
Control	0.04±0.01	0.000		
Model	1.00±0.10			
7 Log-L LVBH107	0.64±0.11	0.000	0.001	
7 Log-HK LVBH107	0.93±0.09	0.264	0.001	
8 Log-L LVBH107	0.89±0.08	0.101		0.240
8 Log-HK LVBH107	0.97±0.09	0.601		0.240

*p*1 is the *p* value compared with the control group, *p*2 is the *p* value compared with 7-L LVBH107 or 7-HK LVBH107 group, *p*3 is the *p* value compared with 8-L LVBH107 or 8-HK LVBH107 group.

Table S9. Effect of *L. mesenteroides* LVBH107 on *IL-6* mRNA expression in RAW264.7 cells.

Group	mean ± standard deviation	*p*1	*p*2	*p*3
Control	0.09±0.01	0.000		
Model	1.00±0.14			
7 Log-L LVBH107	0.54±0.11	0.000	0.010	
7 Log-HK LVBH107	0.85±0.09	0.048	0.010	
8 Log-L LVBH107	0.77±0.09	0.060		0.015
8 Log-HK LVBH107	0.97±0.13	0.633		0.015

*p*1 is the *p* value compared with the control group, *p*2 is the *p* value compared with 7-L LVBH107 or 7-HK LVBH107 group, *p*3 is the *p* value compared with 8-L LVBH107 or 8-HK LVBH107 group.

Table S10. Effect of *L. mesenteroides* LVBH107 on *IL-1β* mRNA expression in RAW264.7 cells.

Group	mean ± standard deviation	*p*1	*p*2	*p*3
Control	0.03±0.01	0.000		
Model	1.00±0.13			
7 Log-L LVBH107	0.86±0.08	0.028	0.048	
7 Log-HK LVBH107	0.98±0.11	0.775	0.048	
8 Log-L LVBH107	0.93±0.05	0.251		0.358
8 Log-HK LVBH107	41.82±2.35	0.769		0.358

*p*1 is the *p* value compared with the control group, *p*2 is the *p* value compared with 7-L LVBH107 or 7-HK LVBH107 group, *p*3 is the *p* value compared with 8-L LVBH107 or 8-HK LVBH107 group.

Table S11. Effect of *L. mesenteroides* LVBH107 on *COX-2* mRNA expression in RAW264.7 cells.

Group	mean ± standard deviation	*p*1	*p*2	*p*3
Control	0.06±0.01	0.000		
Model	1.00±0.07			
7 Log-L LVBH107	0.63±0.04	0.000	0.000	
7 Log-HK LVBH107	0.93±0.08	0.097	0.000	
8 Log-L LVBH107	0.85±0.06	0.020		0.013
8 Log-HK LVBH107	0.96±0.05	0.364		0.013

*p*1 is the *p* value compared with the control group, *p*2 is the *p* value compared with 7-L LVBH107 or 7-HK LVBH107 group, *p*3 is the *p* value compared with 8-L LVBH107 or 8-HK LVBH107 group.

Table S12. Effect of *L. mesenteroides* LVBH107 on *iNOS* mRNA expression in RAW264.7 cells.

Group	mean ± standard deviation	*p*1	*p*2	*p*3
Control	0.11±0.03	0.000		
Model	1.00±0.07			
7 Log-L LVBH107	0.79±0.06	0.001	0.010	
7 Log-HK LVBH107	0.93±0.08	0.146	0.010	
8 Log-L LVBH107	0.86±0.07	0.010		0.014
8 Log-HK LVBH107	0.99±0.051	0.855		0.014

*p*1 is the *p* value compared with the control group, p2 is the *p* value compared with 7-L LVBH107 or 7-HK LVBH107 group, *p*3 is the *p* value compared with 8-L LVBH107 or 8-HK LVBH107 group.
